# Unravelling Plant Responses to Stress—The Importance of Targeted and Untargeted Metabolomics

**DOI:** 10.3390/metabo11080558

**Published:** 2021-08-22

**Authors:** James William Allwood, Alex Williams, Henriette Uthe, Nicole M. van Dam, Luis A. J. Mur, Murray R. Grant, Pierre Pétriacq

**Affiliations:** 1 Environmental and Biochemical Sciences, James Hutton Institute, Errol Road, Invergowrie, Dundee DD2 5DA, UK; 2 School of Earth and Environmental Sciences, The University of Manchester, Oxford Road, Manchester M13 9PT, UK; alex.williams-4@sheffield.ac.uk; 3 Department of Animal and Plant Sciences, Biosciences, The University of Sheffield Western Bank, Sheffield S10 2TN, UK; 4 German Centre for Integrative Biodiversity Research (iDiv) Halle-Jena-Leipzig, Molecular Interaction Ecology Group, Friedrich-Schiller University Jena, Puschstr. 4, 04103 Leipzig, Germany; henriette.uthe@idiv.de (H.U.); nicole.vandam@idiv.de (N.M.v.D.); 5 Institute of Biological, Environmental and Rural Sciences (IBERS), Edward Llwyd Building, Aberystwyth University, Aberystwyth SY23 3DA, UK; lum@aber.ac.uk; 6 Gibbet Hill Campus, School of Life Sciences, The University of Warwick, Coventry CV4 7AL, UK; m.grant@warwick.ac.uk; 7 UMR 1332 Fruit Biology and Pathology, Centre INRAE de Nouvelle Aquitaine Bordeaux, University of Bordeaux, 33140 Villenave d’Ornon, France; 8 Bordeaux Metabolome, MetaboHUB, PHENOME-EMPHASIS, Centre INRAE de Nouvelle Aquitaine-Bordeaux, 33140 Villenave d’Ornon, France

**Keywords:** metabolomics, plant pathology, plant–insect interactions, biotic stress, abiotic stress, targeted, untargeted, systems biology, stress resistance, breeding

## Abstract

Climate change and an increasing population, present a massive global challenge with respect to environmentally sustainable nutritious food production. Crop yield enhancements, through breeding, are decreasing, whilst agricultural intensification is constrained by emerging, re-emerging, and endemic pests and pathogens, accounting for ~30% of global crop losses, as well as mounting abiotic stress pressures, due to climate change. Metabolomics approaches have previously contributed to our knowledge within the fields of molecular plant pathology and plant–insect interactions. However, these remain incredibly challenging targets, due to the vast diversity in metabolite volatility and polarity, heterogeneous mixtures of pathogen and plant cells, as well as rapid rates of metabolite turn-over. Unravelling the systematic biochemical responses of plants to various individual and combined stresses, involves monitoring signaling compounds, secondary messengers, phytohormones, and defensive and protective chemicals. This demands both targeted and untargeted metabolomics approaches, as well as a range of enzymatic assays, protein assays, and proteomic and transcriptomic technologies. In this review, we focus upon the technical and biological challenges of measuring the metabolome associated with plant stress. We illustrate the challenges, with relevant examples from bacterial and fungal molecular pathologies, plant–insect interactions, and abiotic and combined stress in the environment. We also discuss future prospects from both the perspective of key innovative metabolomic technologies and their deployment in breeding for stress resistance.

## 1. Introduction

Climate change and population growth pose a major global challenge to environmentally sustainable food production. Current agricultural challenges include the unsustainable carbon footprint of artificial fertilizers and pesticides, with growing public concern about their environmental and health impacts. Superimposed on this, yield enhancements, through crop breeding, are declining (below 1% year-on-year), while agricultural intensification continues to be constrained by emerging, re-emerging, and endemic pests and pathogens, which are responsible for 20–40% of crop losses worldwide (FAO: http://www.fao.org/news/story/en/item/1187738/icode/ (accessed 15 July 2021)). Climate change will further exacerbate these global issues as rising temperatures affect the geographical distribution of food production, change pest and pathogen distribution, and escalate the magnitude and frequency of abiotic stresses. Thus, feeding an increasing population, whilst limiting land use and environmental impact, represents one of the most significant challenges of the 21st century. It thus becomes increasingly important to deploy innovative genetic and metabolic GWAS (genome-wide association studies) approaches, to define agronomically important QTLs (quantitative trait loci), and adopt new breeding technologies to improve elite crops, focusing on robust disease, pest, and abiotic stress-resistant plants, with lower dependence on chemical fertilizers, while maintaining high yield and quality.

Plants face a multitude of abiotic (e.g., drought, salinity, heat, and water stress) and biotic (pathogenic bacteria, fungi, viruses, nematodes, and insect pests) stresses throughout their life cycles. Plants have evolved a remarkable range of mechanisms to respond rapidly to environmental changes. They can sense the presence of pests, parasites, and pathogens, through evolving effective molecular recognition mechanisms and the capacity to respond to physical changes [1]. The responses to pests and pathogens are complex, involving host–microbe/pest communication and distinct defensive mechanisms. Aside from the physical barriers, innate immune responses, initiated by tissue damage or recognition of microbe/pathogen/nematode-associated molecular patterns—DAMPs, MAMPs, PAMPs, and NAMPs (e.g., lipopolysaccharide—LPS, flagellin, and nematode specific pheromones, i.e., ascarosides)—by host membrane-localized pattern recognition receptors (PRRs), leads to microbe/pathogen-triggered immunity (M/PTI) [2,3]. Adapted pathogens have evolved virulence strategies, deploying proteinaceous or small-molecule “effectors” (virulence factors), which collectively act to suppress PTI and mobilize host nutrients for pathogen nutrition. In some cases, plants have evolved disease resistance (R) proteins that recognize effectors (avirulence factors), or their activity, to initiate a hypersensitive response (HR) that contains the invading pathogen, known as effector-triggered immunity (ETI) [3,4]. ETI also induces systemic-acquired resistance (SAR), a priming response in the whole plant that stimulates long-lasting immunity to a diverse range of pathogens [5,6,7].

The initial stress response of plants is through an integrated network of signaling molecules, inclusive of a wide range of secondary messengers (calcium, reactive oxygen species (ROS), nitric oxide (NO), and lipids) and phytohormones (e.g., salicylic acid (SA), jasmonic acid (JA), abscisic acid (ABA), and ethylene) [7,8,9,10,11]. The network is primarily mediated by mitogen and calcium-activated protein kinase (MAPK and CAPK) signal transduction cascades [12,13,14]. Understanding the timing, amplitude, and duration of signaling events during plant stress, as well as the interplay between phytohormones and transcriptional re-programming, leading to physical and chemical defenses, is a massive, but critically important, challenge within plant biology. Additionally, different subcellular organelles are known to play unique and pivotal roles in integrating biotic and abiotic stress signals, with, for example, chloroplasts orchestrating the production of a range of phytohormones and secondary metabolites [7,15]. This highlights the future importance of sub-cellular metabolomic analyses.

**Figure 1:**
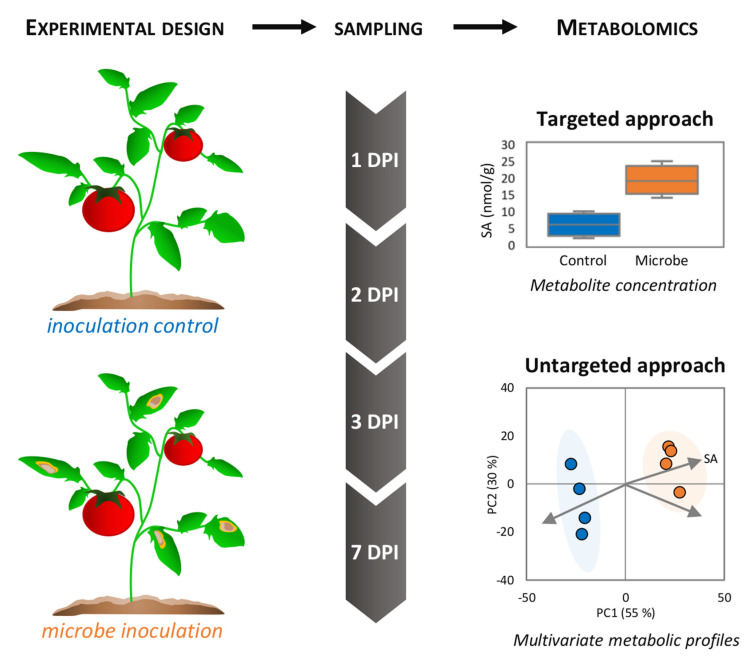
Experimental design in phytopathological metabolomics. Plant tissues (here, tomato leaves) are inoculated without (i.e., mock) or with the pathogenic microbe (here, a fungus causing necrotic lesions), or exposed to other biotic challenges (e.g., elicitors). Subsequent sampling of control and infected material is usually performed following a time course expressed as day post-inoculation (DPI), sample metabolism is quenched by snap-freezing in liquid nitrogen, followed by storage at −80 °C until metabolomic analyses. Targeted metabolomics will allow measuring the levels of specific compounds (here, the defense hormone salicylate, SA). On the other hand, untargeted approaches can evaluate the global impacts of the microbial interactions on metabolic profiles using multivariate statistics (here, a principal component analysis score plot), where the best discriminators could be revealed (represented as arrows, e.g., SA).

**Figure 2:**
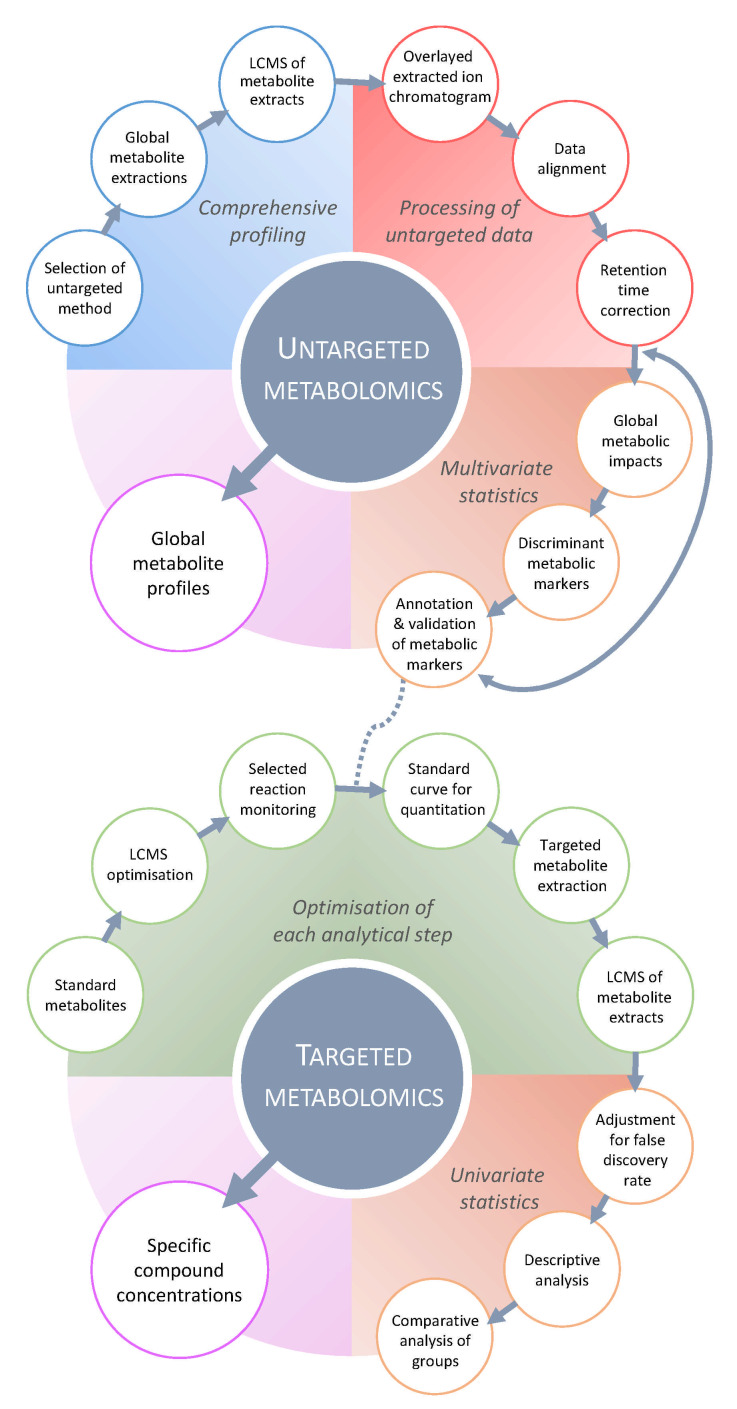
Typical LC-MS-based metabolomics workflow. Targeted metabolomics (bottom panel) aims to deliver quantitative concentrations of specific compounds using metabolite standards to optimize the extraction and LC-MS acquisition. Then, univariate statistical methods allow investigating the targeted data for statistical significance using adjustment of false discovery rate and descriptive analysis (e.g., adjusted *p*-value, single- and multiple-factor ANOVA), and comparative analysis between the control and infected samples at different time points using single or multiple groups (e.g., *t*-test, Kruskal–Wallis, ANOVA with post hoc tests). When using isotopically labelled standards, targeted metabolomics provide absolute quantification of the metabolite of interest. Alternatively, untargeted metabolomics (top panel) generates a comprehensive metabolic overview of several thousands of metabolic features from a global extraction, which are analyzed by untargeted LC-MS profiling, for instance. The processing of untargeted data relies on sophisticated bioinformatics workflow (including extraction, alignment, grouping and retention time correction) for all the detected LC-MS features. Multivariate statistical methods are subsequently applied to the dataset to visualize how metabolic profiles respond to the conditions (i.e., biotic stress) and unravel stress-responsive discriminant metabolic markers. These relevant markers are putatively annotated using metabolite databases, then confirmed by comparing MS/MS and retention time data with metabolite standards, thereby returning to a targeted approach for verification. Targeted and untargeted metabolomics are complementary in addressing how relevant metabolites and metabolism respond or comprehensively react to stress. Adapted from Williams et al. (2020).

Over the past two decades, the emergence of metabolomics as a systems biology strategy has dramatically improved our knowledge of complex biological systems. Similarly to other “omics” sciences, metabolomics has made a prominent contribution to our understanding of plant immune responses [16]. A typical experimental design (Figure 1) includes targeted and untargeted metabolomics strategies, depending on the objective of the study, as follows: (i) the quantification of specific metabolites, due to their importance during the stress response, or (ii) the acquisition of global metabolic profiles from which a search for defense response/resistance metabolic pathways, novel signaling molecules, or biomarkers can be conducted (Figure 2). In the face of microbial challenges, metabolomics has revealed specific and common metabolic signatures as hallmarks of infection processes. These include antimicrobial metabolite counterattacks or metabolic signals, to reorganize plant physiology and adapt to stress. Here, we will examine the major metabolomics techniques and approaches that have made, or have the potential to make, significant contributions to our understanding of plant stress responses, with a specific focus on bacterial, fungal, and pest interactions. We will further overview the metabolomics research into biotic stress responses within the natural environment, and discuss some challenges and perspectives of phytopathological metabolomics.

## 2. Plant Stress Responses Are Metabolically Diverse and Require a Suite of Technologies for Accurate Characterization

Chemical defense underpins survival strategies in the plant kingdom. The interactions between plants and their pathogens and herbivores have led to the evolution of an extensive diversity of defense compounds, representing many chemical classes, including terpenes, alkaloids, and glucosinolates. Defense responses in plants are dynamic, in terms of both the concentration and chemical modification of metabolites. In turn, pests and pathogens evolve new strategies to dampen host defense signaling and overcome the toxic effects of defense compounds, including evolving remarkable bioactive mimics, such as the phytotoxin coronatine, produced by certain strains of *Pseudomonas syringae* [17,18]. Signaling pathways, lipids and phytohormones, ROS generation, structural defenses, such as cell wall strengthening (callose deposition) and the deposition of leaf waxes, all add to this rich chemical tapestry and the technically challenging nature of measuring the plant stress response.

### 2.1. Initial Stress Responses Involve Complex Protein Kinase Signalling Networks and the Generation of Phytohormones

As the precursor to chemical defense, it is of crucial importance to monitor signaling processes in response to stress. MAPK and CAPK cascades are among the first steps downstream of PRR stress/pathogen recognition, and are subsequently deployed during the R protein recognition of intracellular effectors (avirulence factors). These processes are conserved and equally important in response to abiotic stress, such as drought and salinity [19,20,21]. Phosphorylation of the M/CAPK results in the activation of various effector proteins in the cytoplasm or nucleus, including other kinases, enzymes, cytoskeletal proteins, or transcription factors [19,20]. If the protein kinase target is known, its level of activity can be determined by applying Western blotting [22], whereas if the target is unknown, large-scale protein arrays can be screened for phosphorylation activity, in a high-throughput manner [21]. These targets help infer the signaling processes underpinning the metabolic changes that are induced during the plant immune or stress response.

Phytohormones are hugely significant, not just to plant stress responses, but with respect to all aspects of plant growth and development, inclusive of ABA, SA and derivatives, JA and derivatives, the auxins, brassinosteroids, cytokinins, gibberellins, strigolactones, ethylene, NO, pipecolic (PIP) and n-hydroxy pipecolic (NHP) acids, peptide hormones, and polyamines. Phytohormones serve as local and long-distance signaling molecules, and they can act both antagonistically and synergistically, altering defense-related gene expression and transcription levels, cellular division, and growth, both at the localized site of stress and systemically [23]. The phytohormones are generally low-molecular-weight (MW) compounds that provide activity at very low concentrations, 10^−6^ to 10^−5^ mol/L, their turnover rates can be extremely rapid, and once extracted, may be readily degraded, thus providing an analytical challenge. The chemical diversity of phytohormones further adds to this challenge, with phytohormones ranging from being highly polar to non-polar lipids, as well as varying in volatility. Certain hormones, such as SA and JA, are non-volatile, but the simple addition of a methyl group (i.e., MeJA and MeSA) confers volatility, allowing them to act as airborne signals, to communicate to neighboring leaves or plants that they are under attack by herbivores or pathogens. Targeted absolute-quantitative methods, applying ultra-high-performance liquid chromatography (UHPLC) and electrospray ionization (ESI) triple-quadrupole mass spectrometry (QqQ-MS) in conjunction with surrogate-labelled internal standards, which cover a wide range of phytohormones, and from as little as 20 mg of fresh leaf tissue, have been successfully developed [24]. Volatile and semi-volatile phytohormones may also be analyzed via gas chromatography (GC)-MS analysis of methyl-chloroformate derivatives [25]. Ethylene and NO, being extremely low MW gases, require specialist approaches, such as targeted GC–flame ionization detection (FID) [26] or photo-acoustic laser spectrophotometry [27]. Pipecolic and hydroxy pipecolic acids have been determined via GC-MS [28] and liquid chromatography (LC)-MS) [29]. Within samples collected at the localized site of infection or herbivore attack, the levels of phytohormones, such as SA and JA, are induced at levels that are high enough to permit their detection through untargeted LC-ESI-MS or DI/FI (direct infusion/flow infusion)-ESI-MS metabolomics approaches [27,30,31,32]. However, with respect to the long-term priming of SAR, their concentrations are maintained at much lower levels, and therefore targeted LC-QqQ-MS methods are valuable [24].

### 2.2. Redox Carrier/Activation Signals Play a Key Role in the Homeostasis of Reactive Oxygen Species That Lead to Programmed Cell Death

Redox carrier/activation signals, including various pyridine nucleotides (i.e., nicotinamide adenine dinucleotide (NAD) and adenosine triphosphate (ATP)), are also of much importance in ROS and redox signaling against biotrophs and necrotrophs, and the activation of JA responses [33,34]. Likewise, NO has a pivotal role in regulating ROS [8,35]. ATP, NAD+, NADH, and NADPH have classically been assayed via enzymatic colorimetric assays [34,36], although LC-MS and nuclear magnetic resonance (NMR) methods are also available [37,38]. The methods of NO analysis have included the use of fluorescent probes, the Griess reaction, electron paramagnetic resonance (EPR), chemiluminescence, the oxyhemoglobin (Hb-O2) assay, membrane inlet-MS, amperometric methods, and laser photoacoustics [39]. Other important signaling compounds that modulate the expression of genes that are involved in plant signaling, transcriptional regulation, hormone biosynthesis, ROS generation, and polyamine metabolism, include the following: the 3′-phosphoadenosine 5′-phosphate (PAP) pathway, which interacts with ABA, especially during abiotic stress responses (e.g., stomatal regulation in drought and water stress), and which can be determined via HPLC-fluorescence or LC-MS approaches [40]; the non-proteinogenic amino acids, ƴ- and β- amino butyric acids (GABA and BABA), which are involved in defense priming responses to pathogens, insects, and abiotic stresses [41,42], and which can be determined via LC-MS, GC-MS, or NMR; and lipid-derived signals that are liberated from the phospho- and galacto-lipids, both enzymatically and non-enzymatically, including linolenic and linoleic acids, 18 carbon fatty acids that serve as precursors to 12-oxo-phytodienoic acid (OPDA), a C18 cyclopentenone that is converted to JA and its derivatives [30,43], as well as phosphatidic acids (PA’s), which have been associated with the regulation of both SA’s and JA’s [44]. LC-MS lipidomics approaches present a powerful tool for the study of lipid signaling in plants. In conjunction with measuring stress-related and redox activation signals, it is also important to consider ROS generation via histochemical staining, or quantitative determination through the luminol chemiluminescence assay, and the levels of cell death via electrolyte leakage assays [34,45]. Genetically encoded ROS reporters are increasingly being deployed, to provide spatial–temporal information on subcellular ROS generation [46]. A range of genetically encoded reporters, with different subcellular locations, capable of measuring ATP [47], monitoring intracellular H_2_O_2_ dynamics [48], NAD redox status [49], or dynamic changes in NADPH [50], are being developed, and these will be invaluable in providing a spatial context to targeted metabolomics approaches.

### 2.3. Analysis of Central Metabolism, a Range of Defence Compound Chemistries, and Secondary Cell Wall Strengthening, Are Key to Successful Studies of Plant Pathogen or Herbivore-Induced Stress

Signaling events and phytohormonal regulation are, of course, just the tip of the ‘plant stress’ iceberg. Integrated studies, monitoring gene transcript expression, applying proteomics to monitor enzymatic regulation, as well as metabolomic analysis to study defense chemicals, physiological responses, such as cell wall strengthening, and the production of osmotic protectants, will be key to our systematic understanding of a given plant stress response [34,51,52]. Innate defenses are observed against both biotrophic and necrotrophic pathogens, as well as herbivores, and involve generalized physical defenses. The deposition of long-chain leaf cuticular waxes is a common response, both in the defense against herbivore feeding [53] and egg deposition, and with respect to desiccation resistance in abiotic stress [54]. Secondary cell wall strengthening, by the deposition of callose, serves to restrict the invasive spread of pathogens [34,52], and, in some cases, limits the effects of abiotic stresses, such as salinity [55]. Callose, a polysaccharide consisting of β-1,3-glucan with some β-1,6-glucan branches, is commonly deposited in cell walls at the site of the pathogen ingress and is often imaged through fluorescence microscopy. Both the structural changes and spatial distribution of callose can be assessed through approaches such as Fourier transform infrared (FT-IR) and Raman microscopy, or MS imaging (MSi), such as matrix-assisted laser desorption/ionization (MALDI)-MS, desorption (D)ESI-MS, laser ablation (LA)ESI-MS, and time-of-flight secondary ionization (ToF-SI)MS [56,57]. Changes within the structure of callose, in response to stress, could also be assessed through multistage LC-MS/MS and MS*^n^*, as well as 2D NMR post purification. Long-chain cuticular waxes have been classically analyzed with GC-MS [58,59] and, recently, ToF-SIMS has also been applied to the spatial analysis of leaf cuticular wax distributions [60].

Dependent upon the host plant species under investigation, as well as the nature of the biotic stress (and in some examples, abiotic stress), a range of plant secondary metabolite classes, with known toxicity against microbes and/or insects, are present and should be considered for analysis. Classically, glucosinolates and isothiocyanates are important defensive compounds in Brassica responses to herbivory [31,61], as are alkaloids in a range of species, especially the Solanaceae [62,63] and benzoxazinoids, for example DIMBOA (2,4-dihydroxy-7-methoxy-1,4-benzoxazin-3-one) in cereals such as maize [64]. With respect to both microbial pathogens and insect herbivores, phenolic compounds, such as the cyanogenic glycosides [65,66,67], flavonoids [68,69,70], and lignins [71,72], play important roles, as do carotenoids, in relation to stress response signaling and abiotic stress [73], and terpenes, which serve as volatile stress signals that prime neighboring leaves and plants [74,75]. All of these classes are amenable to untargeted metabolomics approaches, with C18 reverse-phase (RP) UHPLC high-resolution (HR)MS, with the exception of the terpenes and carotenoids. Terpenes require volatile amenable methods, such as thermal desorption (TD) or solid-phase micro extraction (SPME) interfaced with GC-MS [74], or, alternatively, proton-transfer reaction (PTR)-MS [75]. Carotenoids require targeted UHPLC-atmospheric pressure chemical ionization (APCI)-MS methods, applying C30 RP columns and high-strength non-polar gradients [76]. Finally, central metabolism, assayed via GC-MS of methoximated trimethylsilyl (MOX-TMS) derivatives, hydrophobic interaction LC (HILIC)-MS, or ^1^H-NMR, is also of importance. Pathogens and insects target photosynthate and carbon storage forms, such as starch, and, in response, plant hosts mobilize primary metabolite resources away from the localized site of attack. Central metabolite hubs, such as phenylalanine, lysine, and glutamate, which divert resources to defensive compounds, play significant roles [77]. Moreover, the importance of osmoprotectants, such as a number of sugars, sugar alcohols, and amino acids, are becoming increasingly recognized, especially in abiotic stress [78,79].

This review has, so far, highlighted that studying plant stress metabolism is a technological challenge, requiring an array of extraction, purification and separation methods, and analytical technologies. To capture the full spectrum of key compound chemistries necessitates a range of conventional tissue extracts, volatile capture methods, and spatial imaging approaches. It is also challenging with respect to the complexity of sub-cellular (e.g., chloroplast) orchestrated defenses, and the differentiation of host and pathogen metabolomes [80]. Here, technologies such as cellular fractionation, cell sorting, and differential isotopic labelling, will be of significant value. In the following sections, we appraise the challenges and provide a review of metabolomics approaches in the field of plant stress, as well as discussing how metabolomics approaches can be deployed to improve our systematic understanding of plant stress and breed improved disease- and stress-resistant crop varieties.

## 3. Metabolomics Research in Molecular Plant Pathology—Challenges and Complexity

It is essential to recognize that, despite the wealth of technical approaches that have been described above, significant biological challenges to studying plant–pathogen interactions still persist. For example, defining the timing of in vivo sampling, to capture the critical early stages of pathogenesis, is one such difficulty. Pathogen effectors work largely in collaboration and in a cell-autonomous manner, hence non-responding tissue invariably consists of the majority of the sample, attenuating biological signal to noise. Furthermore, the bioactive molecules underpinning early responses often do not exhibit large changes in abundance and/or vary dynamically. For example, in any given infected leaf, the establishment of fungal and oomycete haustoria are rare in the overall context of total leaf tissue/cell counts. When sampling entire leaves, this would mean that only the stronger metabolic changes can be elucidated. Though obvious symptoms develop, and pathogen biomass increases later in the infection process, immunity has already been suppressed. Besides, haustorial establishment is asynchronous, thus temporal dynamics are not captured. By contrast, bacterial infections, particularly those that are apoplastically localized, such as *P. syringae* pv. tomato (*Pst*), provide more tractable systems for both targeted and untargeted defense metabolomics. Not only can one alter the inoculum concentration, but synchronous infections across an entire leaf can be established with high-resolution sampling [81]. Moreover, the availability of type III secretion-deficient mutants, where virulence effector delivery into the cell is blocked, provides a readout of the metabolic changes associated with PTI. Thus, bacterial pathosystems can provide insight into the early metabolite changes captured between a non-pathogenic challenge and a virulent challenge, which are modulated by effectors. Despite this, the actual number of infection sites in the whole leaf remains low, even following a high inoculum challenge. Later in the infection process, as the bacterial load increases, metabolomics approaches are most likely capturing changes that are associated with primary metabolism reprogramming, to facilitate pathogen nutrition. Local and distal communication, imperative in defining disease outcome, is predominately facilitated by small molecules. Untargeted metabolomics provides the unparalleled opportunity to uncover new signaling molecules, but will always be constrained by the great diversity and number of chemical structures in plants.

Metabolomics approaches have revealed unexpected levels of complexity, with respect to phytohormonal regulation and balance in biotic stress responses. Traditionally, SA and JA are associated with positively regulating biotrophic and necrotrophic interactions, respectively, and they are also assumed to be mutually antagonistic. The application of transcriptome informed targeted phytohormone analyses, however, identified a critical early role for ABA, revealing that biotrophic pathogens hijack ABA, to promote virulence [82,83]. It is becoming increasingly clear that regulatory changes affecting the bioactivity of phytohormones also need to be analyzed. The conjugation of sugars and amino acids to phytohormones generate a diversity of active and inactive compounds. For example, SA is modified by SA glucosyltransferase enzymes, which are members of the uridine diphosphate (UDP)-dependent glycosyl transferases (UGTs) family. UGT74F1 catalyzes the addition of a glycoside to SA, to form SA–glycoside (SAG) [84]; UGT74F2 catalyzes the formation of the SA glucose ester (SGE); [85] and UGT71C3 catalyzes the conversion of the volatile MeSA to MeSA glycoside [86].

Defense metabolomics evolve. PIP, a lysine catabolite, was originally identified as a key long-distance signaling molecule for SAR [87]. Subsequently, NHP was demonstrated to be the bioactive inducer [88]. Further studies revealed that its signaling function was more complex with glycosylated derivatives implicated [89]. Independent targeted and untargeted metabolomics strategies [90,91] identified UGT76B1 as a glucosyltransferase that modifies bioactive NHP, by catalyzing the formation of 1-O-glucosyl-pipecolic acid. Interestingly, UGT76B1 can also multitask, using SA and dihydroxybenzoic acid derivatives as substrates [92], implicating a pivotal role in modulating immune signaling.

Even with a genetically amenable pathosystem, analytical method development is complex. This is probably best exemplified by the jasmonate family. It was only relatively recently that (+)-7-iso-jasmonyl-L-isoleucine was recognized as the actual bioactive jasmonate [17]. Indeed, jasmonates exist as a particularly large range of derivatives, which contribute positively or negatively to plant defense. The recent development of a two-phase extraction, coupled to LC-MS, for the absolute quantification of multiple jasmonates and the semi-quantitative determination of associated jasmonate pathway metabolites [93], represents major progress in this area.

Metabolomics approaches have also indicated a key role for nucleotides in multiple defense responses. Plant extracellular adenosine-5-triphosphate (eATP) is a DAMP [94]. This recognition of damaged self is consistent with the reported release of cytoplasmic ATP following wounding, leading to apoplastic eATP levels of ~40 uM [95]. Further, eATP is recognized in Arabidopsis by the L-type lectin receptor kinases P2K1/DORN1 (does not respond to nucleotides 1) and P2K2. The activation of these PRRs induces membrane depolarization, Ca^2+^ influx, and ROS formation, conferring enhanced immunity against bacteria, necrotrophic fungi, and herbivores [96,97,98,99]. Paralleling a DAMP role for ATP, the exogeneous application of NAD^+^ or NADP^+^ can also induce both local and systemic pathogen resistance, underpinned by elevated SA and pathogenesis-related gene (PR) expression. Similarly to eATP perception, eNAD^+^ or eNADP^+^ appear to be recognized by lectin receptor kinases [100,101]. The establishment of pyridine nucleotides in activated PTI responses leads to one of the most significant recent discoveries in the field of plant immunity, which is the demonstration that the TIR (Toll-like, interleukin-1 receptor) N-terminal domains of plant resistance proteins (TNLs) undergo proximity-induced dimerization, generating functional NADase enzymatic activity, which is essential for the activation of ETI [102]. The plant TIRs utilize NAD/P^+^, generating a variant cyclic ADP ribose (v-cADPR) with similar mass and mass spectra as cADPR, but a different retention time. Notably, human TIR domains also dimerize to form functional NADases, but their products are ADPr and cADPR. A significant immediate metabolomics challenge is to better understand this “v-CADPR” and the near-identical derivates that are produced by bacterial TIRs [102,103]. Thus, we are now entering a new area, where the absolute quantitation of low-abundance (deoxy)ribonucleotides and (deoxy)ribonucleosides, and their derivatives, such as v-cADPR, will be critical to dissecting the complex plant defense metabolome. This will be greatly facilitated by a new method to quantify nucleotide and nucleoside metabolism in plants [38].

It is quite remarkable that, despite our relatively comprehensive knowledge of the molecular mechanisms underpinning PTI and ETI, and a developing understanding of how pathogen effectors drive effector-triggered susceptibility (ETS), we still do not have a clear understanding of the primary metabolic reprogramming that is associated with pathogen nutrition, let alone know the key metabolites that are utilized by pathogens, as fundamental carbon and nitrogen sources. Clearly, sugar and amino acid metabolism are modified, but space considerations preclude detailed discussion. Instead, we provide two contrasting examples of the effector modulation of primary metabolism for pathogen nutrition. The activation of GABA metabolism has long been associated with plant–microbe interactions. While it has the capacity to provide rich C and N sources, GABA appears to have contrasting effects, depending on the pathosystem under study (reviewed: [104]). An elegant study recently demonstrated that the *Ralstonia solanacearum* effector Rip interacts with host glutamate decarboxylases (GADs), promoting their interaction with calmodulin, to enhance GABA production [105]. In contrast, the indirect hijacking of host metabolism is elegantly illustrated by the activation of host sugar transporters by pathogen effectors. For example, *Xanthomonas oryzae* pv. oryzae, the causal agent of rice bacteria leaf streak, induces (*Oryzae sativa*) clade III “sweet transporters” (OsSWEET11–15), to promote virulence by supporting the translocation of sugars (OsSWEET11 transports sucrose) to the apoplast [106].

Integrating different technologies in a systems level approach will greatly improve our understanding of plant defense responses. Untargeted metabolomics will play a critical role in generating a large compendium of features that are significantly different in diseased or resistant tissues, relative to their appropriate control. Integrating transcriptomics and immunity mutants into such studies can help refine subsets of features to study further. We have already briefly discussed the challenges of signal to noise, from the perspective of disease and defense. While there is a potential scope to deploy cell/tissue-specific markers, sampling remains a challenge, and is further confounded by different pathogen virulence mechanisms and lifestyles. For example, while *Pst* forms discrete apoplastic colonies associated with mesophyll cells, many *Xanthomonas* spp. are vascular pathogens, reflecting the importance of developing methods for spatial sampling. The application of untargeted metabolomic approaches, to investigate stomatal immunity [107], is a recent example of such evolving spatial sampling methods.

## 4. Metabolomics Research in Fungal and Oomycete Plant Pathology

Metabolomic approaches are now well established in the assessment of plant pathogenic fungal and oomycete interactions, and have been extensively considered elsewhere [16,108]. These studies have revealed similar changes in metabolism as previously described in this review, although, unsurprisingly, each discrete plant–fungal/oomycete interaction shows distinctive features. These features have been classified into changes in the following three broad major biochemical groups: alkaloids, isoprenoids, and shikimates [16,109]. Exemplar interactions include alkaloid changes in responses to *Fusarium gramininum*, isoprenoids to *Magnaporthe oryzae*, and shikimates to *Puccinia coronta* [109]. However, metabolomic analyses of fungal- and oomycete–plant interactions represent a considerable challenge, due to the complex spatio-temporal changes that we have already referred to. For example, fungal and oomycete pathogens will often be dispersed via asexually or sexually produced spores, to germinate on the plant surface, and may germinate to form discrete infection structures, which are initially deployed against only a few cells. The key examples are outlined below.

*M. oryzae*—the causal agent of rice blast disease—can infect a range of cereal and grass hosts [110]. *M. oryzae* spore contact on a hydrophobic plant surface leads to germ-tube development and the formation of a melanized appressorium. Metabolite processing, of glycerol for example, with the appressorium, builds up enormous turgor pressures that contribute to the forcing of a penetration peg through the plant cuticle. After penetration, the primary invasive hyphae fill the cell, but still form an intact biotrophic interfacial complex (BIC) with the host, which is vital to the delivery of effectors [111]. Infection hyphae will move to neighboring host cells via plasmodesmata, and ultimately trigger a switch to a necrotrophic mode of pathogenesis [112]. Although phylogenetically distinct, oomycetes exhibit similar infection strategies. With the oomycete *Phytophthora infestans*, the dispersal of asexual zoospores leads to encystment on the plant surface. Cyst germination leads to the formation of an appressorium, penetration through the epidermis (occasionally via the stomata), and the formation of a haustorium, when effectors begin to be delivered. Further hyphal development leads to the formation of additional haustoria in cells in close proximity with the leaf [113,114]. Necrotrophic fungi tend to have less-sophisticated infection strategies, often focusing on the production of toxins and cell wall-degrading enzymes [115]. An important example here is *Fusarium graminearum*, which causes Fusarium head blight (FHB) on a range of cereal crops [116]. This species can secrete a series of mycotoxins, with the most well characterized being deoxynivalenol (DON). It should also be recognized that defense reactions exhibit spatial differences with the formations of cell wall appositions, as well as single or multicellular cell death being displayed at the infection site [117].

*M. oryzae* interactions have been well characterized using metabolomics approaches. Studying *M. oryzae* appressoria formation on a synthetic hydrophobic surface revealed ceramide processing as an important feature of appressorial maturation, linked to the protein kinase C-mediated cell wall integrity pathway [118]. Focusing on plant responses, three hosts of *M. oryzae* showed common changes in amino acids and sugars [110], which likely reflect nutritional reprogramming towards the host [109]. A more comprehensive approach, integrating GC-MS/MS, LC-MS/MS, and ^1^H-NMR assessment of the different responses of rice to virulent and avirulent *M. oryzae* strains, revealed a significant correlation between alanine and disease development, potentially aiding infection by triggering host cell death [119]. By considering temporal changes, the metabolic “conflict” between pathogen nutrient mobilization and host defenses was revealed. During the early stages of infection, there were changes in bioenergetic, polyamine and oxidative metabolism, which is indicative of a defense response, but by the stage of lesion formation, changes in mannitol and glycerol were evident, possibly supporting fungal growth [110]. Resistance to *M. oryzae* may be associated with wide-ranging phospholipid processing, leading to the production of JA, as occurs in the model grass *Brachypodium distachyon* [30]. Genetic evidence in rice has also suggested the importance of JAs, as well as SA and ethylene, in resistance [120].

The *Phytophthora infestans*–tomato interaction is a good example where the transcriptome and metabolomes have been integrated, to derive a robust infection model [121]. Predictive genome-scale metabolic models (GEMs) were derived from *P. infestans* enzyme sequences, which were related to KEGG metabolic networks [122]. This GEM was then improved by incorporating further information, such as organellar location and, crucially, transcriptomic and metabolomic data [121]. These models showed how *P. infestans* growth was dependent on host thiamine biosynthesis. Moreover, separate consideration of the host and oomycete transcriptomic data showed how the pathogen increasingly shuts down some of its own biosynthetic pathways, becoming more dependent on host-sourced nutrients. Although undoubtedly challenging, such multi-omic approaches offer the possibility of major new insights.

Due to the multigenic nature of defense against necrotrophs, metabolomics is a powerful tool that can generate infection biomarkers or, through the use of metabolite (m)GWAS approaches [123], accelerate the breeding of resistant varieties [124,125]. LC-MS has been used to define metabolites associated with a QTL that is linked to resistance against *F. graminearum* in barley [125]. The FHB1 QTL was linked to DON detoxification and, through metabolomics, also to changes in phenylpropanoid and JA metabolism [126]. In wider studies, metabolites linked to constitutive and induced resistance against head blight infections, have been characterized. With DON, the possible biomarkers for this mycotoxin included changes in JAs and the fatty alcohol dihydro-7-hydroxymyoporone [127]. Importantly, responses to the mycotoxin trichothecene were linked to indole acetic acid, picolinic acid, and feruloyl glucosides [128]. *Botrytis cinerea* is an important necrotrophic disease of, particularly, soft fruits, where the identification of biomarkers in presymptomatic (“latent”) infected fruit could help mitigate product spoilage. Recently, untargeted GC-MS approaches identified hexadecanoic acid, octadecanoic acid, sucrose, β-lyxopyranose, melibiose, and 1,1,4a-Trimethyl-5,6-dimethylenedecahydronaphthalene changes in the strawberry latent phase [129].

To address the issue of spatial differences, our early research demonstrated the use of FT-IR microscopy in Arabidopsis, following challenge with *Botrytis cinerea*. Using a series of ethylene defense mutants, imaging at the infection site defined cell wall changes linked to ferulate [27]. More powerfully, MSi approaches can be used where samples are scanned across a predefined field and the intensity of analyte ions is generated at a single point. This generates a pixel reflecting analyte intensity, which can be assembled to form a heat-map image [130,131]. An elegant application of MSi technologies included the use of MALDI-MS [132], to reveal changes in the distribution of glucosinolates in Arabidopsis, which influenced the feeding patterns of cotton bollworm (*Helicoverpa armigera*) larvae [133]. Both FT-IR and MALDI-MSi cannot be easily applied to living tissue, but this is not the case for LAESI-MS. Studying the interaction of *Cladosporium fulvum* on tomato, LAESI-MS provided spatial information on the fungal toxic glycoalkaloid α-tomatine, which suggested its degradation at the site of the infection [134]. Metabolite imaging approaches have yet to be widely applied to plant pathogen interactions, but their potential impact in understanding metabolomic changes could be enormous.

## 5. Metabolomics Research in Plant—Pest Resistance Breeding

Despite all the phytosanitary measures taken by farmers, crops commonly will also be confronted with insect herbivores. It is estimated that ~15% of all crop production is lost to insect herbivore damage annually [135]. Synthetic pesticides have been used to effectively reduce pest pressure and increase crop productivity since the 1950s. The current concerns on the non-target effects of synthetic pesticides, amongst others on pollinating insects, have led to the ban of several effective pesticides, such as neonicotinoids [136]. In addition, several herbivore species have developed pesticide tolerance. This has created the urge for more sustainable herbivore control measures. These strategies go hand in hand with a stronger ability of the plants to defend themselves. Plant breeders are thus left in the unenviable position of balancing the production of novel, pathogen, and pest-resistant varieties that are both tolerant to changing climate conditions and appropriate for the food market.

Metabolomics, combined with bioassays and genetic analyses, is a rapid method to identify specific leads for breeding efforts where natural resistance to pests is paramount. To identify (new) sources of chemical resistance, the large-scale panel screening of existing accessions and wild relatives, using bioassays, is a powerful tool [137]. With a focus on subsets with contrasting levels of resistance, metabolomic analyses can identify specific compounds whose concentrations differ between the susceptible and the resistant accessions. For example, an LC-MS-based metabolomics approach was used to pinpoint dimer acyclic diterpene glycosides (capsianosides) as resistance factors against thrips in Capsicum [53]. In addition, a GC-MS-based metabolomics approach was used to analyze which wax components were related to thrips resistance [53]. For plant breeders, linking these resistance traits to genetic markers is essential. The common approaches are QTL analysis or GWAS mapping [138]. With GWAS, natural accessions are genotyped by sequencing. These same accessions are subjected to metabolomics, which can be combined with QTL maps, yielding information on the genomic regions that certain metabolites, or mQTL, are associated with. Resistance factors have been identified by relating the gene sequence with the metabolome of the individuals [138]. Both mapping methods combined with metabolomics will identify accessions that are associated with resistance and particular metabolic traits. Only by further genetic fine mapping, the responsible regulatory or biosynthetic genes can be identified [138,139]. In addition, these approaches could be combined with targeted genome editing tools, such as CRISPR-Cas9, which precisely target specific DNA sequences [140]. Combined with bioassays, targeted mutations can provide proof-of-principle on the function of specific genes and metabolites. High-throughput metabolomic analyses can identify such desired metabolites in the breeding stock, and thus can be part of other speed-breeding approaches [141], to quickly yield natural insect-resistant accessions. One issue, however, is that plant defenses are often important determinants of the palatability and nutritional value of produce and fodder. Depending on the compound chemistry, plant defense may either be health-beneficial or toxic. Therefore, most crops have been selected to reduce toxicity and bitterness, to increase palatability. A famous example is the reduced concentrations of the glucosinolate progoitrin in oil seed rape. In mammals, progoitrin negatively effects thyroid metabolism, thus causing health issues [142]. The trade-off, however, is that low progoitrin accessions become more susceptible to herbivores [143]. Metabolomic analyses, combined with bioassays, may help to optimize the levels of chemical defenses in crops, without decreasing the nutritional value and marketability of the produce.

## 6. Unravelling the Response of Plants to Stress in the Natural and Agricultural Environment

As plants are adapted to surviving a multitude of climatic extremes over their growth season, which often occur simultaneously, within close proximity or at multiple points during development, their metabolic response needs to be plastic [144]. With increasingly frequent weather extremes in agriculturally valuable areas, understanding the metabolic underpinnings of the plant response to environmental stress is an important avenue for safeguarding natural and managed ecosystems [145,146]. Due to the vast suite of primary and secondary metabolites that plants employ to aide in combined (a)biotic stress responses, developing and employing metabolomics technologies is imperative to discern the interactive impacts on system level metabolomics, and highlight potential agricultural exploitation for stress mitigation strategies, as discussed in the previous section.

Targeted metabolomics allows relative or absolute quantification of individual, or a small number, of metabolites. For example, phytohormones are often measured using targeted approaches [24,25]. Considering future CO_2_ levels, GC–chemical ionization (CI)-MS revealed that reduced JA production in maize, at elevated CO_2_ (eCO_2_), coincided with reduced phytoalexin production and increased susceptibility to Fusarium wilt [147]. Similarly, HPLC-fluorescence was used to show that increases in SA, at eCO_2_, coincided with increased scopoletin levels and resistance to *Pst* in Arabidopsis [148]. Interestingly, phytohormonal changes under eCO_2_ link to changes in the structure of the secondary metabolome, which impacts the immune response. In eCO_2_, untargeted GC-MS revealed that changes in the primary metabolite profile overlapped with increases in an oxidative stress background, and thus highlighted a role for redox metabolism of the plant during CO_2_ stress [148]. Similarly, untargeted UHPLC-ESI-MS was used to show that plants that were grown in low CO_2_ had a secondary metabolite profile that was consistent with greater ROS production, oxidative stress, and resistance to biotrophic pathogens [149]. Low CO_2_ altered the photorespiration machinery, with knock-on impacts on the sugar metabolism and antioxidant status of the plant. This also suggests that photorespiration played a greater role in immunity in past CO_2_ climates.

Metabolomics has found substantial application in drought research too. From directed studies, such as GC demonstrating that mannitol accumulates in the roots of droughted olives [78], to targeted LC-MS methods, illustrating major changes in many primary and secondary pathways, including amino acid metabolism and flavonoid biosynthesis in JA mutant backgrounds [150]. In drought-tolerant sesame, untargeted GC-MS identified accumulation of ABA, proline, arginine, lysine, GABA, saccharopine, 2-aminoadipate, and allantoin [151], many of which are implicated and are of importance in plant–microbe responses. In this case, by coupling metabolome and transcriptome data, a strong argument was demonstrated that ABA itself is likely altering metabolite profiles, to generate a more osmotically stable environment. This is complementary with ABAs role in drought-induced stomatal closure, increased stomatal defense [152], and it serving as a direct signal in the rhizosphere, to recruit drought-protective microbes [153]. Combining different omics approaches is a powerful tool for better understanding and unravelling complex tripartite interactions. For instance, combined transcriptome and metabolome data, measured via LC-MS, in Arabidopsis experiencing combined drought, warming and viral infection, indicated that signaling networks shifted in a stress-specific manner, and resulted in a breakdown in plant defenses under multi-stress [154]. Further, the impact of heat stress can impact the pathogen response, by requiring a contrasting metabolomic strategy, rendering the plant more susceptible to disease [155]. In maize, the metabolomes under heat stress and disease were shown to be divergent, using both untargeted and targeted UHPLC-MS. In a combined stress scenario, hydroxycinnamic acid and p-coumaric acid accumulation were specifically identified to contribute to the heat-induced susceptibility of maize to the necrotrophic leaf pathogen *Cochliobolus heterostrophus* [155]. Previous examples illustrate that individual stresses are inadequate for determining the mechanisms of high-temperature-induced disease susceptibility [156], which is likely true across all (a)biotic stresses that require contrasting plant responses.

The benefits of the application of metabolomics technologies, to investigate complex tripartite stress interactions, are clear, although challenges remain. Future innovation will permit an integrated view of changes in dynamic plant metabolomes, across the tissue spectrum of the plant, in natural field systems with specific dissection of host and microbial metabolomic responses to a suite of environmental stresses.

## 7. Future Prospects: Which New Tools and Approaches Will Make an Impact?

Metabolomics is a valuable tool for discovering metabolic signals for which the compound identification procedure is underprovided and tedious [157]. Advances in bioinformatics have dramatically improved metabolomic data processing [158,159], but, despite this, thousands of signals remain unidentified [160]. Thus, metabolomics could have a much greater impact, with the development of improved annotation workflows and more comprehensive plant metabolite libraries. Machine learning now plays a significant role in metabolomics (LC-MS being routinely utilized [161]). It predicts a vast array of molecular properties and processes, such as adduct species, MS fragmentation, MS/MS deconvolution, and chromatographic retention time [158,159,162]. However, it must still be noted that, due to the vast numbers of closely related isomeric compounds in plants, as well as their derivatives decorated with varying isomeric sugar moieties or fused with amino acids, which produce near-identical MS/MS and MS*^n^* fragmentation patterns, and which co-elute even with long (60 > min) UHPLC separations, there needs to be a focus upon developing higher resolution separation methodologies and technologies that can be combined with both MS and 2D NMR. The development of 2D LC separations, as well as time-series retention index standards that are applicable to LC, as n-alkanes and fatty acid methyl esters (FAMES) are applied in GC, would provide a massive step change towards developing retention time (RT)-MS/MS and MS*^n^* libraries that are transferable between different LC-MS systems and laboratories. Overcoming such hurdles and enhancing our metabolite annotation capabilities will be particularly valuable to the discovery of antimicrobial and viral molecules in plant–pathogen research. A recent study reports the metabolic setting of plant–virus interactions in a natural habitat and its predictive link to susceptibility, unravelling new insight into plant–virus interactions and providing attractive antimicrobial targets [163]. Likewise, machine learning-based predictive metabolomics is particularly promising, to reveal and validate metabolic predictors under biotic stress [164]. Future bioinformatics developments for metabolomics will reduce human intervention and increase data analysis throughput, thanks to robotized assays, for instance [157,164].

As previously mentioned, MSi is prominent among the new tools that have been developed for metabolome analysis [165], and offers promising perspectives for the spatial dissection of metabolic responses to infection, although without chromatographic separation it does lack the ability to differentiate structural isomers that display identical MS/MS and MS*^n^* fragmentation patterns. However, by applying ion mobility separations prior to HRMS detection, there is the potential to resolve isomeric compounds, without prior chromatographic separation. Recent salient MSi examples include the barley defense metabolite serotonin and other metabolites of the melanin pathway against *M. oryzae* [166], and the sulfur-containing glucosinolates in Arabidopsis, which intervene in fungal and pest responses [133]. Besides MSi, other approaches allowing the isolation of cells and organelles (e.g., flow cytometry, cell and organelle sorting, microfluidics) will make an outstanding contribution to the future impacts of metabolomics on plant stress studies.

Genome-scale metabolic modelling also holds great promise to study plant–pathogen interactions. This computational systems biology perspective implies structural and comparative genomics, transcriptomics, and protein–protein interactions [167]. The calculation of metabolite coefficients for genome-scale metabolic flux predictions requires high-resolution metabolome data (easily provided by today’s Orbitrap and Q-TOF technologies) and high-resolution temporal sampling, as emphasized by a typical experimental design of phytopathological metabolomics (Figure 1). In this context, genome-scale metabolic models of potato and tomato leaves and *Phytophthora* have simulated the metabolic fluxes during infection [121,168]. Interesting molecular aspects, involving photosynthesis suppression by the pathogen, were demonstrated, as well as the uptake of nutrients by *Phytophthora* following the infection cycle phases. It should be noted that stage-specific profiles of the joint metabolism of the host and pathogen could be refined by integrating high-resolution data from the tomato infection metabolome [121].

**Figure 3:**
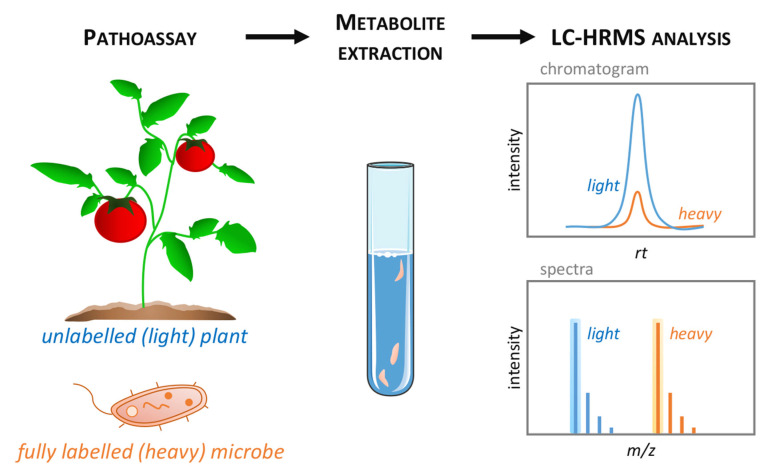
LC-HRMS discrimination between native and labelled metabolites. Unlabelled (light) plant tissues (e.g., leaves) are inoculated with fully labelled (heavy) pathogenic microbe (e.g., ^13^C isotopically labelled bacteria) then sampled at the appropriate time point for metabolome analysis (left). The resulting metabolite extracts thus contains a mixture of light and heavy biochemical (middle) that could be discriminated by LC-HRMS technique (right). Light and heavy metabolites display similar retention time (RT) on the LC-HRMS chromatogram, but natural isotope clusters of light and heavy forms are well separated on the mass spectra.

To echo this last point, one of the main challenges of metabolomics in plant pathology is distinguishing plant and microbial metabolomes [80]. Metabolites, most particularly primary compounds, are often ubiquitous in the diverse plant, microbial and animal organisms, or exchanged between the host and pathogens, as signals or metabolic responses. In pathological studies (Figure 1), plants and microbes are jointly incubated, but the pathogens are rarely removed before metabolite extraction, thereby mixing metabolomes of different origins. This probably seems to be of minor importance for other omics, such as transcriptomics and proteomics, which exploit species-specific databases. However, metabolomics suffers from cross-contamination of the host and non-host metabolites; therefore, it is often difficult to draw precise conclusions as to the origin of the metabolic variations found. Whilst one can search data for metabolites of non-plant origin, for example, arachidonic acid with respect to fungal pathogens, one cannot assume that their absence suggests that metabolites of fungal origin are not being detected. Implementing stable isotope labelling of microbes could address this pressing concern. A recent metabolomics study of the early stage of plant cell–microbe interactions employed an incubation method of ^13^C- and ^15^N-labelled (98%) *Pst* DC3000 with Arabidopsis cells, followed by a washing step to remove the excess bacterial cells [169]. Subsequently, “light” plant metabolites were analyzed by targeted LC-MS, highlighting an infection reprogramming of plant signaling and primary metabolic pathways, including carbohydrates, nucleotides, and amino and organic acids. Alternatively, uniformly ^13^C- and ^15^N-labelled plants can be prepared, which will offer an accurate tool for metabolomic analysis under biotic stress conditions [170]. The growing interest in isotopically labelled biological material coincides with the development of additional analytical tools, such as NMR and high-throughput GC- and LC-HRMS, which allow the discrimination between native and labelled metabolites (Figure 3). It is conceivable that elegant studies of this type will be carried out less sporadically, to help clarify the origin of changes in the host and pathogen metabolome. In summary, future prospects that will greatly contribute to the success of metabolomics in the study of plant immunity will include (i) increasing the capacity to chromatopgraphically resolve and annotate plant and pathogen metabolomes, (ii) new tools and approaches to analyze the metabolome at the subcellular level, and (iii) the development and implementation of modelling, to predict and/or calculate the dynamics of metabolic variations.

## 8. Concluding Remarks

Metabolomics has revolutionized the understanding of plant–microbe interactions, by providing a valuable biochemical phenotypic screen of the chemical diversity of plants and their metabolic responses to biotic challenges. Targeted approaches have permitted a better insight into the role of key molecules that are involved in these phytopathological mechanisms, such as phytohormones, and other defense and signaling biochemicals. Untargeted metabolomics has also pivotally unveiled new and unsuspected metabolic players, such as pyridine cofactors and ATP, for instance. However, the use of metabolomics in the study of plant–microbe interactions still lags other omics approaches, notably transcriptomics. This problem is even more imperative for plant–virus interactions, for which there has been very little research. At a time when humanity is suffering from an unprecedented pandemic of COVID-19, the study of the metabolic pathways that are involved in viral resistance, as well as the discovery of plant antiviral molecules, has a bright future [162]. Beyond metabolite profiling, the forthcoming trend is likely towards more holistic and comprehensive large-scale multi-omics strategies, critically providing necessary insight at the systems level [171]. A closer association between metabolomics and chemical ecology will also facilitate discovering a plethora of metabolites with ecological and defensive roles [172].

## Abbreviations

^1^H-NMR	proton nuclear magnetic resonance spectroscopy
2D NMR	two-dimensional nuclear magnetic resonance spectroscopy
ABA	abscisic acid
ADP	adenosine diphosphate
APCI	atmospheric pressure chemical ionization
ATP	adenosine triphosphate
BABA	β-amino butyric acid
cADPR	variant cyclic ADP ribose
CAPK	calcium-activated protein kinase
CI	chemical ionization
CO_2_	carbon dioxide
DAMP	damage-associated molecular patterns
DESI	desorption electrospray ionization
DI	direct infusion
DON	deoxynivalenol
eATP	extracellular adenosine-5-triphosphate
*e*CO_2_	elevated carbon dioxide
EI	electron ionization
EPR	electron paramagnetic resonance
ESI	electrospray ionization
ETI	effector-triggered immunity
ETS	effector-triggered susceptibility
FAMES	fatty acid methyl esters
FHB	Fusarium head blight
FI	flow infusion
FID	flame ionization detection
FT-IR	Fourier transform infrared
GABA	ƴ-amino butyric acid
GAD	glutamate decarboxylase
GC	gas chromatography
GEM	genome-scale metabolic models
GWAS	genome-wide association studies
HILIC	hydrophobic interaction liquid chromatography
HPLC	high-performance liquid chromatography
HR	hypersensitive response
HRMS	high-resolution mass spectrometry
JA	jasmonic acid
LAESI	laser ablation electrospray ionization
LC	liquid chromatography
LPS	lipopolysaccharide
MALDI	matrix-assisted laser desorption ionization
MAMP	microbe-associated molecular patterns
MAPK	mitogen-activated protein kinase
MeJA	methyl jasmonic acid
MeSA	methyl salicylic acid
MOX-TMS	methoximated trimethylsilyl
mQTL	metabolite quantitative trait loci
MS	mass spectrometry
MS/MS	tandem MS
MSi	mass spectrometry imaging
MS*^n^*	multi-stage mass spectrometry
MTI	microbe-triggered immunity
MW	molecular weight
NAD	nicotinamide adenine dinucleotide
NADH	nicotinamide adenine dinucleotide + hydrogen
NADPH	nicotinamide adenine dinucleotide phosphate
NAMP	nematode-associated molecular patterns
NHP	n-hydroxy pipecolic acid
NMR	nuclear magnetic resonance spectroscopy
NO	nitric oxide
OPDA	12-oxo-phytodienoic acid
PA	phosphatidic acid
PAMP	pathogen-associated molecular patterns
PAP	3′-phosphoadenosine 5′-phosphate
PIP	pipecolic acid
PRR	pattern recognition receptors
*Pst*	*Pseudomonas syringae* pv. Tomato
PTD	pathogen-triggered defense
PTI	pathogen-triggered immunity
PTR	proton transfer reaction
QqQ	triple-quadrupole mass spectrometry
QTL	quantitative trait loci
Q-ToF	quadrupole time-of-flight
R protein	resistance protein
ROS	reactive oxygen species
RT	retention time
SA	salicylic acid
SAG	salicylic acid glycoside
SAR	systemic acquired resistance
SIMS	secondary ionization mass spectrometry
SPME	solid-phase micro extraction
TD	thermal desorption
TIR	Toll-like, interleukin-1 receptor
TNL	N-terminal domains
ToF	time-of-flight
UHPLC	ultra-high-performance liquid chromatography
v-cADPR	variant cyclic ADP ribose
